# Systematic Review of Longitudinal Evidence and Methodologies for Research on Neighborhood Characteristics and Brain Health

**DOI:** 10.3389/phrs.2024.1606677

**Published:** 2024-03-26

**Authors:** Yvonne L. Michael, Araliya M. Senerat, Channa Buxbaum, Ugonwa Ezeanyagu, Timothy M. Hughes, Kathleen M. Hayden, Julia Langmuir, Lilah M. Besser, Brisa Sánchez, Jana A. Hirsch

**Affiliations:** ^1^ Department of Epidemiology and Biostatistics, Dornsife School of Public Health, Drexel University, Philadelphia, PA, United States; ^2^ Urban Health Collaborative, Dornsife School of Public Health, Drexel University, Philadelphia, PA, United States; ^3^ Department of Internal Medicine, Medical Center Boulevard, Winston-Salem, NC, United States; ^4^ Department of Social Sciences and Health Policy, Bowman Gray Center for Medical Education, Winston-Salem, NC, United States; ^5^ Department of Neurology, Comprehensive Center for Brain Health, University of Miami Miller School of Medicine, Miami, FL, United States

**Keywords:** longitudinal study, neighborhoods, cognition, adults, aging, Alzheimer’s disease

## Abstract

**Objective:** Synthesize longitudinal research evaluating neighborhood environments and cognition to identify methodological approaches, findings, and gaps.

**Methods:** Included studies evaluated associations between neighborhood and cognition longitudinally among adults >45 years (or mean age of 65 years) living in developed nations. We extracted data on sample characteristics, exposures, outcomes, methods, overall findings, and assessment of disparities.

**Results:** Forty studies met our inclusion criteria. Most (65%) measured exposure only once and a majority focused on green space and/or blue space (water), neighborhood socioeconomic status, and recreation/physical activity facilities. Similarly, over half studied incident impairment, cognitive function or decline (70%), with one examining MRI (2.5%) or Alzheimer’s disease (7.5%). While most studies used repeated measures analysis to evaluate changes in the brain health outcome (51%), many studies did not account for any type of correlation within neighborhoods (35%). Less than half evaluated effect modification by race/ethnicity, socioeconomic status, and/or sex/gender. Evidence was mixed and dependent on exposure or outcome assessed.

**Conclusion:** Although longitudinal research evaluating neighborhood and cognitive decline has expanded, gaps remain in types of exposures, outcomes, analytic approaches, and sample diversity.

## Introduction

Given the combination of a rapidly aging global population [1] and that Alzheimer’s disease (AD) and related dementias (ADRD) remain incurable conditions [2], research efforts have emphasized identifying risk factors or interventions for healthy aging to prevent cognitive decline and maintain cognitive health. Importantly, racial/ethnic and socioeconomic disparities in AD/ADRD exist and are expected to widen [3]. These factors support the importance of detecting effective interventions for subgroups to achieve health equity.

While research has primarily focused on individual-level interventions for maintaining brain health [4, 5], the neighborhood environment’s role in brain health has become a subject of investigation due to its multiple pathways to health [6]. Neighborhoods may provide important opportunities or obstacles for physical engagement, social interactions, and access to interventions or treatments which could enrich or hinder older adults’ lives and buffer against or accelerate cognitive decline. Two recent systematic reviews provide limited evidence that neighborhood resources are moderately protective of cognitive decline among older adults [7, 8]. Specifically, increased green space/park exposure, community size, and better transportation infrastructure are significantly linked to better cognitive health [8]. Conversely, features of the environment may accelerate progression of AD/ADRD by isolating older adults and limiting access to care, eliciting stress, or containing harmful exposures associated with AD/ADRD, such as air pollution [9]. More critically, perhaps due to declining physical and cognitive function and a diminishing social network, community-dwelling older adults are less likely to leave their immediate environment for work or recreation, making them particularly susceptible to neighborhood effects [10]. Notably, the majority of studies encompassed by previous systematic reviews are cross-sectional in nature, considerably limiting the ability to establish causal relationships between cognitive health and neighborhood factors.

As the area of neighborhood and cognition research has progressed, the importance of longitudinal evidence is increasingly recognized. Longitudinal research offers insight into the causal role of environmental exposures by providing temporality and allowing for the assessment of change in neighborhood conditions [11]. Additionally, it sheds light on AD/ADRD-related progression over time and the mechanisms by which specific features of the environment impact onset of cognitive symptoms. Recent debates regarding modeling of longitudinal research illustrate the complexity of methods necessary to optimize our ability to draw causal inference [12]. Neighborhood data are additionally methodologically unique due to geospatial relationships and opportunities for nesting of observed data [11, 13, 14]. Long-standing methodological obstacles, such as the appropriate size and shape of a neighborhood [15–18], have been investigated in neighborhood cardiovascular research but not for research on cognitive health. Despite these complexities, no existing review has provided an in-depth analysis of longitudinal research or synthesized key methodologies used in this growing field.

Neighborhoods play a role in creating and reinforcing racial/ethnic and socioeconomic disparities in brain health. For example, well-documented geographic patterns of neighborhood resources in the United States show increased access to health-supportive features for predominantly white or wealthy areas [19–21]. Yet the field’s ability to investigate the impact of neighborhoods on cognition for specific subgroups or to understand the interplay between neighborhood disparities and AD/ADRD disparities rests on additional methodologic challenges [22]. Few longitudinal studies have adequate population variability to examine differences by race/ethnicity or socioeconomic status [23]. Even studies with variability may not have adequate sample size to assess effect modification with sufficient precision. This may explain why limited attention to date has focused on the potential modifying effect of factors such as race/ethnicity on the association between neighborhood factors and cognition, despite the evident need for this research [24].

This review aims to systematically assess longitudinal research examining associations between neighborhood characteristics and cognitive outcomes in older adults and to identify the methodological approaches used to account for nested data and estimate change. We extract neighborhood exposures, cognitive outcomes, and the methods employed by this growing field. We also emphasize study population characteristics and potential effect measure modification to detect findings relevant to address racial/ethnic and socioeconomic disparities. Through this systematic literature review and synthesis, we strive to identify existing gaps and guide ongoing and future work.

## Methods

### Search Strategy

We searched Ovid MEDLINE, PsycInfo, Web of Science, and Embase through 1 July 2022 and supplemented with a reference list from another systematic review [8] (see Supplementary Table S1 for detailed literature search strategy).

### Study Selection and Data Extraction

**FIGURE 1:**
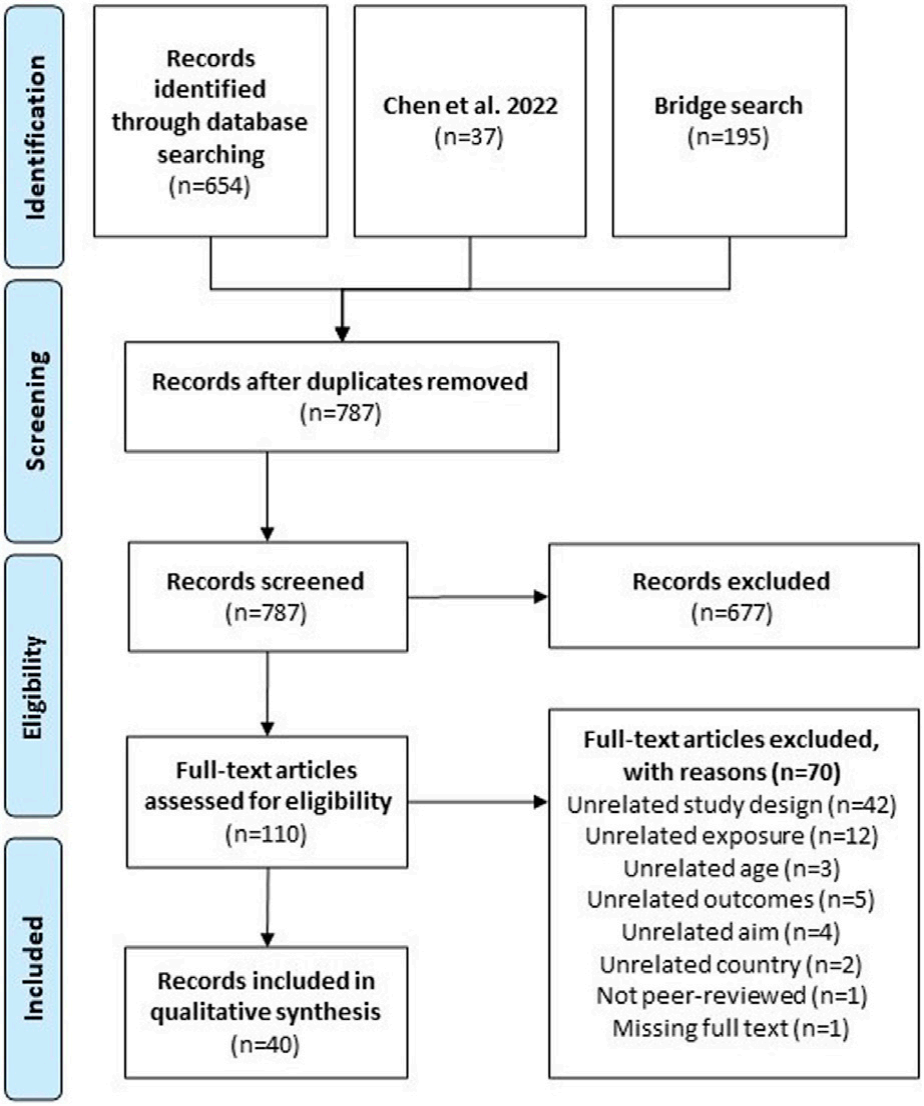
Preferred reporting items for systematic reviews and meta-analyses (PRISMA) flowchart of the systematic literature process (performed Philadelphia, United States. 2022).

Two reviewers independently reviewed 787 unique citations and 110 full-text articles against *a priori* inclusion criteria (Figure 1; Supplementary Table S1). Longitudinal analyses of the effect of built or social neighborhood environment on the cognitive function among community-dwelling participants aged (≥45 years or mean age of 65 years) were included. We excluded articles that evaluated environments outside of neighborhoods (e.g., hospitals; assisted living facilities) and those that only evaluated outdoor air/air pollution or COVID-19 pandemic-related changes. To ensure comparability across countries, we excluded studies conducted in countries rated medium or low on the 2020 Human Development Index (HDI). We extracted data on: country, aim of study, population characteristics, length of longitudinal study, neighborhood exposures, spatial unit of analysis, timing of exposure measurement (once, repeated), brain health outcomes, type of longitudinal approach, method to account for correlation, and effect measure modification. Two reviewers independently abstracted data from each included study with any disagreements resolved by consensus or a third reviewer, if necessary. All selection, screening, and extraction was done using Covidence (Melbourne, AUS).

### Article Data Synthesis and Analyses

After extraction of all relevant information, articles were classified by exposure, outcome, and analysis methods. Neighborhood-related exposure measures were categorized into broader topics (Supplementary Table S2) by two neighborhood health experts (JH and YM). Exposures were categorized as single time-point or multiple time-points. Within papers with multiple time-points, type of longitudinal exposure was analyzed (time-varying; cumulative average; change). Cognition-related outcome variables were categorized with input by two neuro-epidemiologists (TH and KH) and one neuropsychologist (Supplementary Table S3). A combination of study design, details, and methods were used to classify papers by analytic strategy. We identified the analytic approach to the longitudinal outcome data (time to event; repeated measure analysis; difference; autoregressive; risk estimation) by reviewing modeling information including statistical model, type of outcome, and whether and how time was used. Additionally, we considered how correlations in exposure or outcomes were accounted for in analyses using nesting or multi-level clusters (none; clustered within geographic area; clustered within individual; both). Papers were considered to evaluate potential effect measure modification of the association between neighborhood and cognition if they included interaction terms and/or reported stratified estimates. We then classified type of effect measure modification based on effect modifier of interest (e.g., race/ethnicity, gender, socioeconomic status). We calculated frequencies of all exposure, outcome, and analysis categories across all papers. The direction of association was also reported and summarized as showing significance, showing no significance, or mixed results.

## Results

### Included Studies and Populations

The process of study selection is presented as a PRISMA flow diagram in Figure 1. A final count of 40 studies were screened in this literature review. A plurality of the studies were conducted in the United States (43%) [6, 25–40] or China (20%) [41–48] (Supplementary Table S4). Fifty-eight percent of studies included White or European-origin participants [6, 25–35, 37–40, 49–55], 43% included Asian or Pacific Islander participants [25, 26, 33, 36, 41–48, 56–60], and 30% included Black or African American participants [6, 25–32, 34, 39, 40] (Table 1). Half of the studies had only one racial/ethnic background or did not report race [36, 42–50, 52–55, 57–59, 61–63], with only 13% including four or more racial/ethnic groups [25, 26, 31, 34, 39]. Most studies collected environmental exposures through spatial boundaries (47.5%) [6, 25, 28, 29, 34, 35, 38, 46–51, 53, 58–61, 63] or administrative boundaries (37.5%) [26, 27, 30–33, 37, 39, 41, 42, 52, 54, 56, 57, 62], 15% did not specify or used a subjective definition of neighborhood [36, 40, 43–45, 55].

**TABLE 1:** Descriptive of included studies.

Author, year	Country	Total years spanned	Brain health outcomes											
			Clinical diagnosis	Adjudicated diagnosis	MRI Scans	Verbal learning	Memory	Verbal fluency	Executive function	Global cognitive Score	Attention	Cognitive function/Cognitive decline	Dementia	Alzheimer’s disease
Astell-Burt, 2020	Austra-lia	10 to 14		X									X	
Besser, 2021	United States	5 to 9							X	X		X		
Besser, 2022	United States	5 to 9							X	X		X		
Cherrie, 2018	United Kingdom	5 to 9								X		X		
Cherrie, 2019	United Kingdom	5 to 9								X		X		
Clarke, 2015	United States	15+								X		X		
de Keijzer, 2018	United Kingdom	10 to 14					X	X	X	X		X		
Fernández-Blázquez, 2021	Spain	5 to 9		X								X		
Finlay, 2020	United States	5 to 9				X	X	X		X		X		
Finlay, 2021	United States	10 to 14								X		X		
Finlay, 2022	United States	10 to 14								X		X		
George, 2020	United States	15+	X	X									X	
Ho, 2020	China	5 to 9	X										X	
Hsu, 2022	Taiwan	15+								X		X		
Hunt, 2021	United States	10 to 14			X							X		
Liu, 2020	Taiwan	<5		X									X	
Luo, 2019	China	<5								X		X		
Meyer, 2021	United States	5 to 9				X	X		X			X		
Mobley, 2022	United States	15+		X									X	
Motohiro, 2021	Japan	<5								X		X		
Ouvrard, 2020	France	15+		X									X	
Paul, 2020	Canada	10 to 14		X									X	
Peng, 2022	China	5 to 9								X		X		
Rodriguez-Loureiro, 2022	Belgium	10 to 14	X									X		
Sharifian, 2020	United States	5 to 9					X	X				X		
Slawsky, 2022	United States	5 to 9								X			X	
Tang, 2020	United States	<5					X		X	X		X		
Tani, 2019	Japan	<5		X									X	
Vassilaki, 2022	United States	10 to 14		X								X	X	
Wandell, 2020	Sweden	10 to 14		X									X	
Wang, 2022	China	5 to 9		X						X		X		
Watts, 2015	United States	<5					X			X	X	X		X
Weuve, 2021	United States	10 to 14					X		X	X				X
Worn, 2017	Netherlands	5 to 9					X		X	X		X		
Xiong, 2021	China	<5								X		X		
Yu, 2021	United States	5 to 9								X		X		
Yuchi, 2020	Canada	<5		X										X
Zhang, 2022	China	5 to 9					X			X		X		
Zhu, 2019	China	10 to 14								X		X		
Zhu, 2020	China	10 to 14								X		X		

Environmental exposures (X = one time point, L = multiple time points)														
Environmental hazards	Greenery/Greenspace exposure/Blue exposure	Retail food environment	Neighborhood cohesion	Neighborhood disorder/Aesthetics/Quality	Neighborhood Socioeconomic Status (SES)	Neighborhood Segregation	Social destinations	Public transportation	Walkability	Urbanicity/Rurality Status	Infrastructure	Elevation/Hilliness	Healthcare facilities	Recreation/Physical activity facilities
	X													
														X
						X								
														L
														L
				X			X	X	X					
	L													
					X									
		L												
									X					X
X		X					X							X
					L									
	X									X				
										L				
	X				X									X
	X						X							X
X					X		X	X			X		X	X
						X								
					X									
							X			X		X		
					L									
	L													
			X											
	X													
			X	X										
	X													
			L	L										
		X												
					X									
					X									
					X									
			X						X					
L														
					X					X				
				X										
			X	X										
L	L													
	L													
	L													
	L													

Analysis type				Effect measure modification (R = race, S=SES, G = gender, N = none)	Multilevel approach			Racial/ethnic makeup					
Time to event	Repeated measure	Difference/auto	Risk		None	Individual cluster	Neighborhood cluster	Not specified	White or European	Black or African American	Asian Pacific Islander	Hispanic or latino	Other
X				N		X	X	X					
	X			R		X	X		X	X	X	X	
	X			R, S		X	X		X	X	X	X	
		X		G, S			X		X				
		X		G	X				X				
	X			N		X	X		X	X			X
	X			G, S		X			X				X
X				S	X			X					
	X			N		X	X		X	X			X
	X			N		X	X		X	X			X
	X			R, S, G		X	X		X	X			
X				R, S	X				X	X			
			X	N	X						X		X
	X			N		X	X				X		X
	X			N		X			X	X		X	X
	X			N		X					X		
	X			N		X	X				X		
	X			R		X			X	X		X	
X				R	X				X		X		
		X		N	X						X		
X				N	X				X				
X				S, G			X	X					
	X			S, G		X					X		
X				S, G			X		X				
	X			N		X			X	X		X	X
X				N	X				X				X
	X			N		X					X		
X				N	X						X		
X				G		X			X				X
X				G	X				X				
	X			S		X					X		
		X		N	X				X				X
	X			R, S		X			X	X		X	X
	X			N		X			X				
	X			N		X	X				X		
		X		N	X				X	X			X
			X	R, G	X						X		X
	X			N		X	X				X		
	X			G, S		X					X		
			X	N	X						X		

### Neighborhood Exposures and Outcomes

**FIGURE 2:**
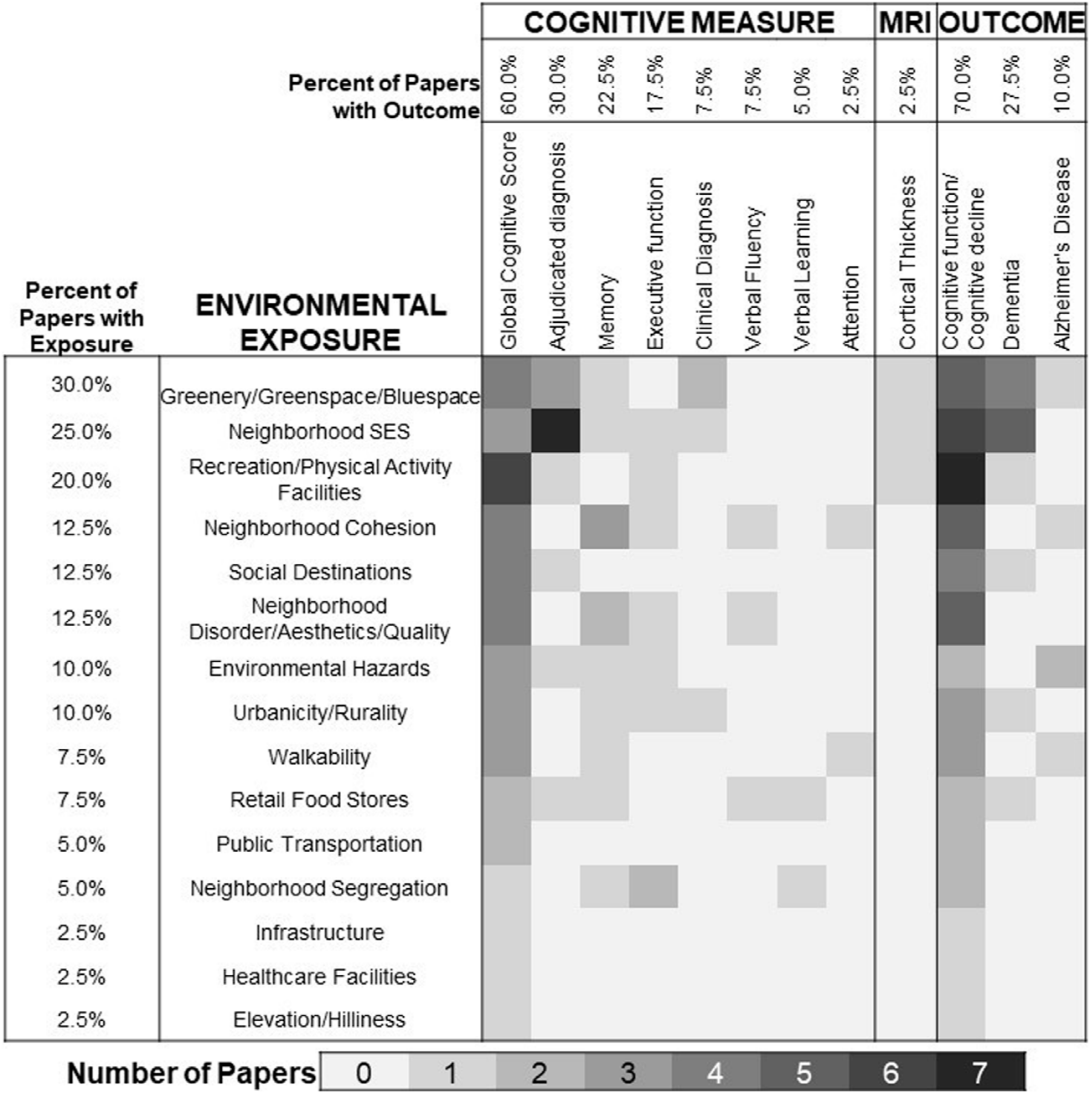
Coverage of environmental exposures and cognitive outcomes in longitudinal research published between 2015 and 2022. Note, cells are not mutually exclusive as many papers examine multiple exposures or multiple outcomes.

We categorized environmental measures into 15 exposure types (Supplementary Table S2). The most common environmental exposures were green space and/or blue space (water) (30%) [35, 41, 46–48, 51, 53, 57, 60, 61, 63], neighborhood socioeconomic status (SES) (25%) [30, 31, 33, 37, 42, 44, 52, 54, 55, 62] and recreation/physical activity facilities (20%) (Figure 2) [6, 25, 29, 42, 49, 50, 57]. Most studies (65%) only provided environmental exposure measures at one timepoint (Table 1) [6, 25–27, 29, 31–35, 37, 38, 40–45, 53–55, 57–59, 61, 62]. Over half of the papers (70%) examined cognitive function or cognitive decline [6, 25–29, 31, 32, 34, 36–38, 40, 42–51, 53, 55, 56, 58, 60, 62], often through global cognitive score (60%) [6, 25–29, 35, 36, 38–40, 42–51, 55, 56, 58]. Very few papers used magnetic resonance imaging (MRI) data (2.5%) [31] or adjudicated diagnosis of AD/ADRD (10.0%) [30, 33, 37, 52, 54, 57, 59–63]. Symbolizing exposure-outcome pairs (Figure 2) illustrated substantial gaps in evidence. For example, while neighborhood cohesion [34, 36, 38, 40, 43], social destinations [6, 27, 42, 57, 58], and neighborhood disorder [27, 34, 36, 40, 45] were not underrepresented in the overall sample (12.5% of studies had each), none of those papers examined MRI outcomes and only one examined dementia [57].

### Analytic Approach to the Longitudinal Brain Health Data

Over half of the studies used repeated measures analysis to evaluate changes in the brain health outcome (52.5%). A little over a quarter of the studies analyzed the length of time until the occurrence of AD/ADRD (27.5%). A little over ten percent of the studies modeled calculated change or used an auto-regressive model to estimate change in brain health (12.5%). The remaining studies (7.5%) estimated risk or odds of an AD/ADRD event without including time in the calculation.

A third of the studies did not account for any type of correlation in analyses through nesting or multi-level clusters (35%). Another third (30%) accounted for within individual correlation in the outcome (e.g., temporal clustering). The remaining third accounted for clustering of individuals within neighborhood (e.g., spatial clustering) with or without accounting for level-one (individual-level) correlation (35%).

### Effect Measure Modification

Less than half (47.5%) of the studies evaluated whether the influence of neighborhood on cognitive outcomes varied by race/ethnicity (20%), SES (30%), and/or gender/sex (*n* = 27.5%). Of the papers that evaluated race/ethnicity, none identified statistically significant effect measure modification [6, 25, 26, 30, 32, 33, 39, 60]. Regarding effect modification by socioeconomic status, while most studies did not find evidence of significant effect modification [6, 26, 30, 43, 47, 50, 51, 53, 62, 63], two studies suggested the influence of neighborhood characteristics on changes in cognition was stronger among low socioeconomic status participants [39, 44]. One study found the association between park availability and brain health was strongest among women [49]. Another study found that the association between residential surrounding greenness and baseline cognition was stronger for men, but the association between greenness and cognitive decline was stronger for women [51]. None of the other studies evaluating effect measure modification identified a statistically significant effect [6, 37, 43, 47, 50, 53, 54, 60, 63].

### Association Between Neighborhood Exposure and Brain Health

The evidence regarding the association between neighborhood exposures and change in brain health is mixed (Table 2). When examining results by type of outcome measure, most analyses that assessed neighborhood factors with MRI scans and verbal fluency displayed an association (100% and 67%, respectively). The exposures most likely to be associated with cognitive change were neighborhood disorder/aesthetics/quality (60%), public transportation (50%), neighborhood cohesion (40%), and social destinations (40%). Among the few studies to examine environmental hazards, walkability, and healthcare facilities, most reported no significant association (50%, 67%, and 100%, respectively). However, the literature investigating neighborhood exposures remains sparse, with few studies evaluating exposures aside from green space/blue space or neighborhood SES.

**TABLE 2:** Direction of evidence by exposure, outcome, and analysis type.

Paper characteristic	N^a^	Mixed	No association	Association
Cognitive Measure				
Clinical Diagnosis	3	33% (1)	33% (1)	33% (1)
Adjudicated diagnosis	11	64% (7)	9% (1)	27% (3)
Verbal Learning	2	50% (1)	50% (1)	0% (0)
Memory	9	44% (4)	33% (3)	22% (2)
Verbal Fluency	3	0% (0)	33% (1)	67% (2)
Executive function	7	43% (3)	29% (2)	29% (2)
Global Cognitive Score	24	46% (11)	29% (7)	25% (6)
Attention	1	100% (1)	0% (0)	0% (0)
MRI Scans				
Cortical Thickness	1	0% (0)	0% (0)	100% (1)
Cognitive Outcome				
Cognitive function/Cognitive decline	29	66% (19)	7% (2)	28% (8)
Dementia	11	45% (5)	18% (2)	36% (4)
Alzheimer’s Disease	3	100% (3)	0% (0)	0% (0)
Neighborhood Exposure				
Environmental hazards	4	50% (2)	50% (2)	0% (0)
Greenery/Greenspace exposure/Blue exposure	10	50% (5)	20% (2)	30% (3)
Retail food environment	3	67% (2)	33% (1)	0% (0)
Neighborhood cohesion	5	60% (3)	0% (0)	40% (2)
Neighborhood disorder/Aesthetics/Quality	5	40% (2)	0% (0)	60% (3)
Neighborhood Socioeconomic Status (SES)	9	33% (3)	33% (3)	33% (3)
Neighborhood Segregation	2	100% (2)	0% (0)	0% (0)
Social Destinations	5	20% (1)	40% (2)	40% (2)
Public Transportation	2	50% (1)	0% (0)	50% (1)
Walkability	3	0% (0)	67% (2)	33% (1)
Urbanicity/Rurality Status	4	75% (3)	0% (0)	25% (1)
Infrastructure	1	100% (1)	0% (0)	0% (0)
Elevation/Hilliness	1	100% (1)	0% (0)	0% (0)
Healthcare facilities	1	0% (0)	100% (1)	0% (0)
Recreation/Physical Activity Facilities	6	33% (2)	33% (2)	33% (2)
Analysis Type				
Time to event	11	55% (6)	18% (2)	27% (3)
Repeated measure	21	71% (15)	5% (1)	24% (5)
Difference/auto	5	60% (3)	20% (1)	20% (1)
Risk	3	33% (1)	0% (0)	67% (2)
Multilevel				
None	14	57% (8)	14% (2)	29% (4)
Individual Cluster	12	67% (8)	0% (0)	33% (4)
Neighborhood Cluster	3	33% (1)	33% (1)	33% (1)
Both	11	73% (8)	9% (1)	18% (2)

^a^ Total may not add up to 40 due to some papers examining multiple characteristics.

## Discussion

This systematic literature review examined studies on associations between neighborhood characteristics and cognitive outcomes and brain MRI in older adults. While cross-sectional evidence is mounting for these relationships and has been reviewed elsewhere [7, 8] our review focused only on studies that examined longitudinal associations between neighborhood and brain health. Across 15 different neighborhood exposures examined in the research we reviewed, most papers focused on three types of environmental exposures (greenspace, neighborhood SES, and recreation/physical activity facilities) and a majority lacked longitudinal exposure data (i.e., measured environment at only one timepoint and had longitudinal cognitive outcomes). This is consistent with the two previous systematic reviews [7, 8]. Similarly, despite reason to believe that neighborhoods may play a role in creating and reinforcing racial/ethnic and socioeconomic disparities in brain health, we found that the existing longitudinal literature has generally ignored these variations in association or had insufficient diversity of sample to test for effect measure modification. Most research selected appropriate statistical models to analyze longitudinal data but a minority of studies used multi-level modeling to distinguish between neighborhood-level and individual-level influences on changes in brain health.

Our review highlights a need for more longitudinal evidence across a comprehensive set of neighborhood features and outcomes. A large proportion of the papers focused on greenery/greenspace/bluespace (30%), neighborhood SES (25%), and recreation/physical activity facilities (20%). This leaves very limited work on other environmental factors and their pathway to cognition, including neighborhood cohesion, social destinations, neighborhood disorder/aesthetics/quality, environmental hazards, urbanicity/rurality, walkability, retail food stores, public transportation, neighborhood segregation, infrastructure, healthcare facilities, or elevation/hilliness. Greenspace and recreation/physical activity facilities are merely two features of the built environment, while theoretical models propose the existence of potentially synergistic effects stemming from additional features such as third places/destinations, density/land use mix, and connectivity/mobility (e.g., public transportation) [6, 64]. These same models emphasize the importance of neighborhood social environments including safety/crime, disorder, and social connections/cohesion, all of which are currently underrepresented in the longitudinal literature. Our finding of the under-representation of social environments as a risk factor for cognitive aging and dementia is consistent with findings from a recent scoping review which called for the inclusion of time-varying social environmental factors and multiple social ecological levels in future research [65]. Interestingly, these social environment measures may also be more consistently associated with cognitive outcomes; our review showed that neighborhood disorder/aesthetics/quality, neighborhood cohesion, and social destinations were three of the top six exposures significantly associated with change in brain health. Additionally, while it is encouraging to see papers examining underlying fundamental causes of neighborhood features, such as neighborhood SES, more work should be done on additional factors that result in unequal resource distribution such as residential segregation by age or race/ethnicity. Since our review, a few studies evaluating segregation have been published which suggest future research is necessary [26, 66]. Similar gaps and opportunities exist for future work to examine additional measures of brain health. Within our review, 72.5% of papers assessed cognitive function or cognitive decline and 60% focused on Global Cognitive Scores. Very few papers examined indicators such as MRI (2.5%), or clinical outcomes such as all-cause dementia (27.5%) and Alzheimer’s disease (7.5%). MRI scans may be less biased by racial/ethnic, language, and socioeconomic aspects in cognitive testing [67, 68]. This observation becomes particularly relevant as the field strives to understand disparities in brain health and the differential impact of neighborhood factors among different subgroups.

This review identified a growing number of longitudinal studies. Longitudinal studies are critical not just for providing temporality, but also for understanding changes in neighborhood conditions and risk for AD/ADRD. However, a minority of these studies employed multilevel modeling to account for the nesting of individuals within neighborhoods. By using multilevel modeling, researchers can estimate the extent to which group-level factors (e.g., neighborhood characteristics) influence cognition or cognitive events, while controlling for individual-level factors [69]. This approach allows for the investigation of both between-group and within-group variability, as well as the examination of how group-level and individual-level variables are related to individual-level outcomes. Multilevel modeling requires an adequate sample size to detect group-level effects; failure to account for the nested data may reflect insufficient sample to appropriately model the neighborhood influences of interest. Researchers need to conduct sample size and power calculations to ensure that there are enough participants and groups to detect the effects of interest [69], and greater resources and funding should target health-related research at the neighborhood level that use spatial sampling frames to ensure adequate sample size. None of the studies specifically referenced sample size considerations related to their modeling approach.

Most commonly the research we reviewed selected appropriate statistical models, such as mixed models, to analyze longitudinal data with continuous outcomes. These models account for the correlation between repeated assessment of the outcome (e.g., cognitive function) within individuals and the nested structure of the data. However, few studies addressed any baseline imbalance in neighborhood-level factors by using appropriate statistical methods, such as analysis of covariance (ANCOVA) or change models [12]. These methods can help control for confounding and improve the accuracy of estimates. However, studies must clearly define the causal estimates of interest, such as etiological research focused on changes in cognitive function or new events, rather than, for example, health services research focused on burden of disease associated with neighborhood characteristics, to guide the choice of appropriate analysis methods [12]. Further, we did not identify any studies that used alternative analysis methods, such as gain scores or graphical models or marginal structural models controlling for time varying confounder, to assess trajectories of change in cognitive function or cognitive events [70–72]. These methods may provide additional insights into the relationships between neighborhood factors and cognitive outcomes.

In our review, more than half of the studies did not evaluate differences in the association between neighborhood and cognition by key social determinants of health, including race/ethnicity, SES, or gender. Similarly, half of the studies used a single-race or ethnic background sample. Research identifying neighborhood features associated with disparities in cognition are important to ensure that future policy and design-based interventions are carefully planned to reduce inequalities rather than exacerbate them [73]. Risk for AD/ADRD is unequally distributed across gender, SES, and race/ethnicity. African American, Hispanic, low-SES individuals face the highest and most disproportionate risk for AD/ADRD [74–80]. These variations in brain health describe high risk populations but fail to identify specific pathways through which to intervene to reduce disparities. Without diverse cohorts and comprehensive evaluation of the impact of systemic and interpersonal racism, we may be missing key contributors of disparities in brain health. The distribution of neighborhood features and resources varies by neighborhood-level SES and racial composition [21, 81–88]. Relative differences in these neighborhood features may be the clue to deciphering racial/ethnic and SES disparities in AD/ADRD. Consistent with our findings, a recent scoping review of neighborhood influences on racial disparities in cognitive health found that even among studies with diverse samples of racial and ethnic groups, few studies evaluated disparities and those that did used inconsistent approaches to evaluating effect modification by race/ethnicity. Future research, including adequate diversity and attention to outcome measures that are resistant to the biases of traditional cognitive testing, is needed to inform both policy-level, population health interventions and clinician-based interventions tailored to individual patients’ circumstances.

This review is not without its limitations. First, gray literature was not included, suggesting that some articles may have been missed in such a rapidly growing field. Similarly, exclusion of non-English studies restricts the overall generalizability and completeness of this review. Additionally, this review only captured studies conducted in high or very high HDI countries. While the purpose of this exclusion was to make findings more comparable across these countries, results may not be generalizable to less developed settings or countries. Finally, many papers examined multiple exposures (or operationalizations of the same exposure) or outcomes. While we have summarized papers overall and summarized evidence by analyses, this complexity hinders our ability to give a concise or conclusive account of the state of the literature. This lack of consistency across both exposures and outcomes also restricted our ability to perform a quantitative synthesis or estimate statistical combinations of results across studies.

Despite these limitations, this review advances the field’s understanding of the measures, methods, and populations currently represented in the longitudinal literature on neighborhood environments and changes in cognitive status. Specifically, we identified exposure-outcome pairs that warrant more examination, catalogued existing analytic methods including use of multi-level modeling or clustering, explored the inclusion of effect measure modification to understand disparities, and provided an initial summary of the strength of the evidence to guide prioritization of exposures or outcomes. Future work should continue to study both individual neighborhood environment measures and holistic measures of the composite impact of interrelated neighborhood features across the lifespan (e.g., exposome). Analyses should leverage longitudinal data through more complex, advanced methods and modeling techniques. To understand, estimate, and address disparities in cognitive decline, new studies should push for more diverse population samples, robust outcome measures, and analysis that explicitly estimate effects across race/ethnicity and socioeconomic status.
