# Supplementary material for: Systematic Review of Longitudinal Evidence and Methodologies for Research on Neighborhood Characteristics and Brain Health
Source: Public Health Rev. 2024 Mar 26;45:1606677. doi: 10.3389/phrs.2024.1606677 (PMC11002187; doi:10.3389/phrs.2024.1606677)
Supplement: Supplementary file 1 [file DataSheet1.DOCX]

**Supplementary Material**

**Supplementary Table S1: Detailed Literature Search Strategy**

| **Database** | **Neighborhood Environment** | **Cognitive Outcomes** | **Older Adulthood** |
| --- | --- | --- | --- |
| Ovid MEDLINE | Residence characteristics/ or housing/ or public housing/ or exp Environment Design/ or ("environmental design" or neighborhood* or neighbourhood* or "built environment" or ((neighborhood* or urban) adj2 (factor* or design* or environment* or character* or level)) or walkabil*).ti,ab,kf. | exp Cognition/ or exp cognition disorders/ or dementia/ or alzheimer disease/ or exp Memory Disorders/ or cognitive dysfunction/ or (alzheimer* or dement* or cognitive function* or "cognition" or "mild cognitive impairment" or ((cognit* or memory) adj2 (defic* or declin* or impair*)) or neurodegener*).ti,ab,kf. | exp Aged/ or exp Aging/ or age factors/ or "age of onset"/ or age factors/ or "age of onset"/ or cognitive aging/ or (aging or ageing or (older adj2 adult*) or elderly).ti,ab,kf. |
| Web of Science | ALL=(Residence characteristic* or environment design or neighbor$rhood* or "built environment" or neighborhood factor* or urban factor or neighborhood design* or neighborhood environment or walkabil*)) | ALL=((cognitive or cognition or dementia or Alzheimer* or demented))) | ALL=((older adult* or elderly or senior citizen* or aged or "later life" or older people))) |
| Embase | 'Residence characteristics'/exp OR 'residence characteristics' OR 'housing'/exp OR housing OR 'environmental planning'/exp OR 'environmental planning' OR 'built environment'/exp OR 'built environment' OR 'neighborhood'/exp OR neighborhood OR 'walkability'/exp OR walkability OR 'green space'/exp OR 'green space' | 'cognition' OR 'cognitive defect' OR 'dementia' OR 'alzheimer disease' OR 'cognitive decline' OR 'mild cognitive impairment' | 'aged' OR 'aging' OR 'older adult' OR 'cognitive aging' OR 'aging' OR 'geriatric disorder' |
| APA PsycINFO | (DE "Communities" OR DE "Urban Environments" OR DE "Environmental Enrichment" OR DE "Social Density" OR DE "Social Ecology" OR DE "Environmental Effects" OR MM "Aging in Place" OR DE "Home Environment" OR DE "Living Arrangements" OR DE "Active Living" OR DE "Communities" OR DE "Neighborhoods")) OR (DE "Built Environment") | (DE "Alzheimer's Disease" OR DE "Senile Dementia" OR DE "Senile Psychosis" OR DE "Dementia" OR DE "Memory Disorders" OR DE "Mild Cognitive Impairment" OR DE "Cognition" OR DE "Cognitive Impairment")) | (DE "Gerontology" OR DE "Geriatrics" OR DE "Geriatric Assessment" OR DE "Geriatric Psychiatry" OR DE "Older Adulthood" OR DE "Cognitive Aging" OR DE "Healthy Aging" OR DE "Physiological Aging") |

**Supplementary Table S2: Neighborhood-related exposure measures categorization**

| **Main Categories** | **Environmental Measures** |
| --- | --- |
| **Environmental hazards** | Living close to main roads/highways |
|  | Road tidiness |
|  | Polluting sites density |
|  | Noise |
| **Greenery/Greenspace exposure/Blue exposure** | Greenery/Greenspace exposure/Blue exposure |
| **Retail food environment** | Retail food environment |
|  | Grocery store density |
|  | Fast-food and coffee density |
| **Neighborhood cohesion** | Neighborhood cohesion |
|  | Sense of community/social connectivity/neighborhood connectivity |
| **Neighborhood disorder/aesthetics/quality** | Neighborhood disorder |
|  | Perceived Neighborhood Safety |
|  | Quality of public spaces/Childhood neighborhood quality |
| **Neighborhood SES** | Neighborhood SES |
|  | Neighborhood disadvantage/Neighborhood SES disadvantage/deprivation |
| **Neighborhood Segregation** | Neighborhood Segregation |
| **Social Destinations** | Social organization density |
|  | Religious organization density |
|  | Senior services density |
|  | Educational organization density |
|  | Arts organization density |
|  | Museum density |
|  | Library density |
|  | Proximity to nearest community center |
|  | Employment service |
| **Public Transportation** | Access to public transit/distance to bus stop |
|  | Bus lines |
| **Walkability** | Neighborhood walkability |
|  | Neighborhood business density |
|  | Integration (how many turns must be made from a street segment to reach other street segments) |
| **Urbanicity/Rurality Status** | Urban compactness/sky view factor/Urbanity |
|  | Rurality |
|  | Population characteristics |
|  | Residential density |
| **Infrastructure** | Basic infrastructures |
|  | Days roads unpassable |
|  | Handicapped access |
| **Elevation/Hilliness** | Elevation |
|  | Hilliness |
| **Health care facilities** | Health care facilities |
| **Recreation/Physical Activity Facilities** | Playgrounds and sport venues |
|  | Outdoor exercise facilities |
|  | Recreation center density |
|  | Neighborhood Park Access |

**Supplementary Table S3: Cognition-related outcome variable categorization**

| **Main Categories** | **Brain Health Measures** |
| --- | --- |
| **Clinical Diagnosis** | Death certificates |
| **Adjudicated diagnosis** | Diagnosis from medical provider/prescriptions/hospital admissions/electronic health records |
| **MRI Scans** | MRI Scans |
| **Verbal Learning** | Word List Learning (WLL) |
| **Memory** | Word List Delayed Recall (WLD) |
|  | Free Recall Test/Short Term Memory |
|  | Semantic memory |
|  | East Boston Memory Test-Immediate Recall |
|  | East Boston Memory Test - Delayed Recall |
|  | Episodic memory |
| **Verbal Fluency** | Animal Fluency Test (AFT)/Animal Names Test |
|  | S words test |
|  | Letter Fluency Test (LF) |
| **Executive function** | Executive function |
|  | Digit Span backwards test |
|  | Alice Heim 4 test of intelligence |
|  | Moray House Test No. 12 (MHT) |
|  | Symbol Digit Modalities Test (SDMT) |
|  | Digit Symbol Coding (DSC) |
|  | Raven Colored Progressive Matrices |
| **Attention** | Attention |
| **Global Cognitive Score** | Global Cognitive Estimate/Function |
|  | Montreal Cognitive Assessment (MoCA) |
|  | Mini-Mental State Examination/Chinese-MMSE (MMSE/C-MMSE)/ Modified MMSE |
|  | CASI |
|  | Global Clinical Dementia Rating (CDR) |
|  | Telephone Interview of Cognitive Status (TICS) |
|  | Cognitive Assessment for Dementia (CADi2) |
|  | Alzheimer's Disease Assessment Scale (ADAS) |
|  | Short Portable Mental Status Questionnaire (SPMSQ) |

**Supplementary Table S4: Summarized Study Characteristics**

| **Study Characteristics** | | **% (N)** |
| --- | --- | --- |
| **Publication Years** | 2015 | 5% (2) |
|  | 2017 | 2.5% (1) |
|  | 2018 | 5% (2) |
|  | 2019 | 10% (4) |
|  | 2020 | 32.5% (13) |
|  | 2021 | 22.5% (9) |
|  | 2022 | 22.5% (9) |
| **Country** | Australia | 2.5% (1) |
|  | Belgium | 2.5% (1) |
|  | Canada | 5% (2) |
|  | China | 20% (8) |
|  | France | 2.5% (1) |
|  | Japan | 5% (2) |
|  | Netherlands | 2.5% (1) |
|  | Spain | 2.5% (1) |
|  | Sweden | 2.5% (1) |
|  | Taiwan | 5% (2) |
|  | UK | 7.5% (3) |
|  | United States | 42.5% (17) |
| **2020 Health Development Index status** | High | 17.5% (7) |
|  | Very High | 82.5% (33) |
| **Total years study spanned** | <5 | 20% (8) |
|  | 5 to 9 | 37.5% (15) |
|  | 10 to 14 | 30% (12) |
|  | 15+ | 12.5% (5) |
| **Exposure: Spatial unit of analysis** | Administrative boundaries | 37.5% (15) |
|  | Spatial and administrative | 2.5% (1) |
|  | Spatial boundaries | 47.5% (19) |
|  | Subjective definition | 12.5% (5) |
| **Number of times environmental exposure was measured** | Once | 65% (26) |
|  | Multiple timepoints | 35% (14) |
| **Number of times cognition was measured** | Once | 12.5% (5) |
|  | Multiple timepoints | 87.5% (35) |
| **Number of different racial/ethnic categories measured** | Not specified | 7.5% (3) |
|  | 1 | 42.5% (17) |
|  | 2 | 25% (10) |
|  | 3 | 12.5% (5) |
|  | 4 | 12.5% (5) |
| **Results presented by different socioeconomic status** | Yes | 30% (12) |
|  | No | 70% (28) |
| **Effect Measure Modification by race/ethnicity, SES, and/or gender** | Yes | 47.5% (19) |
|  | No | 52.5% (21) |
